# Research on the Optimal Allocation of Ecological Land from the Perspective of Human Needs—Taking Hechi City, Guangxi as an Example

**DOI:** 10.3390/ijerph191912418

**Published:** 2022-09-29

**Authors:** Jingheng Wang, Yecui Hu, Rong Song, Wei Wang

**Affiliations:** 1 School of Land Science and Technology, China University of Geosciences, 29 Xueyuan Rd., Beijing 100083, China; 2 Key Lab of Land Consolidation and Rehabilitation, Ministry of Natural Resources of the People’s Republic of China, 37 Guanying Rd., Beijing 100035, China

**Keywords:** ecological land use, human needs, multi-objective planning, PLUS model

## Abstract

The configuration of ecological land directly affects the structure and function of an ecosystem and, ultimately, its ability to meet human needs. From the perspective of human needs, this paper classified human needs into material needs, security needs and spiritual needs. Using Hechi City, Guangxi as the study area, we combined the Multi-objective planning (MOP) and PLUS models to study the quantity and spatial optimization of ecological land under different human needs scenarios, and the optimal allocation of ecological land within the ecological red line was also discussed. We conclude that: (1) Hechi City currently has less arable land, which cannot fully guarantee the material needs of human beings; there is more forest land than the amount needed to meet human needs, which reduces the efficiency of ecological land use. (2) In terms of quantity optimization, and considering the goals of different human needs, the area of grass to forest should be extended to satisfy security needs; the area of arable land should be significantly increased in line with material needs; the area of grass and water, with the goal of accommodating spiritual needs, is the largest compared with the rest of the goals. Under the comprehensive needs goal, the forest land area is greatly reduced, and the rest of the land area is increased; the goals of human material, spiritual and security needs are basically met. (3) In terms of spatial layout optimization, in order to meet the security needs target, grass to forest conversion should be carried out in the northern area to enhance the aggregation of forest land; to meet the material needs target, the southwestern gentle slope area should be concentrated toward continuous farming to guarantee the aggregation of arable land while increasing the area of arable land; to meet the spiritual needs target, the area of water in the northwestern area should be increased, and the rest of the changes are similar to the security needs target; to meet the comprehensive needs target, the overall land use connectivity becomes stronger, the fragmented land types become less and the concentrated continuous area of forest land, arable land and grass increases. (4) The results of the ecological land adjustment within the ecological red line indicate that the current ecological red line delineation is good, and a small amount of adjustment can meet human needs. Based on human demand, combined with the MOP-PLUS model for ecological land optimization, it can accurately portray the spatial and temporal evolution pattern of land use and reveal the optimization path of ecological land, which has important theoretical and practical values.

## 1. Introduction

The process of human civilization is developing in interdependence with the natural environment. Human beings have the social need to grow the economy and enjoy rich material life, but also have an ecological need to be close to nature to maintain the body and mind [1]. The damage to the ecological environment caused by the various needs of human beings in the course of civilization is often drastic, so China proposed to draw a “red line for ecological protection” [2]. The human need for ecological land was ignored when the ecological red line was designated. The area of ecological land is often used as a reference to determine the area of the ecological red line [2,3,4,5]. This has resulted in the unfulfillment of the ecological need to be close with nature in more populated areas, and the inability of less populated areas to meet human social and economic needs due to massive restrictions on the use of ecological land. The relationship between the various types of ecological land is not scientific, and so this leads to the ecological land in the region being unable to meet a variety of human needs at the same time. In this paper, ecological land is studied, and because arable land has both productive and ecological functions, it is included in the discussion of ecological land.

At present, there are four mainstream views on the identification of ecological land in China and abroad. (1) “Ecological element determinism”, which defines ecological land mainly from the perspective of land spatial morphology, believes that the spatial positioning of ecological elements is collectively called ecological land. This is divided into two categories according to spatial morphology: mature forests, lakes and water bodies, wetlands, agricultural land and open space are considered surface ecological land, while rivers and coastal mudflats are considered linear ecological land [6]. (2) “Pan-ecological function determinism”, which defines ecological land purely from the perspective of land ecological function, believes that any land that can provide ecosystem services can be regarded as ecological land, including agricultural land, forest land, grassland, water and swamp, etc., and ground without artificial pavement and permeable surface can be included within the scope of ecological land [7]. (3) “Main function determinism”, which divides ecological land by the main function of land, considers ecosystems that mainly play natural ecological functions and are important for maintaining key ecological processes as ecological land, with important ecosystem service functions and high ecological sensitivity [8]. (4) “Determinism of use form”, which classifies ecological land from the perspective of whether it is exploited or not, and considers land other than agricultural land and construction land that is not used by humans and can perform ecological functions directly or indirectly as ecological land [9].

Changes in ecological lands directly affect the structure and function of ecosystems and, ultimately, their ability to provide ecosystem services. Furthermore, variations in ecosystem services, which form and maintain the basis of the natural environmental conditions on which humans depend, can directly or indirectly affect human well-being [10,11]. Therefore, the scientific delineation of ecological land from the perspective of human needs is essential for promoting human welfare and enhancing the efficiency of ecological land use. At the relationship level between ecological land and human needs, ecological land can produce goods and provide the corresponding services (supplies) needed by humans to meet human needs (needs), which are expected to include food, water, air, internal balance, rest and purification of pollutants [12].

There are multiple definitions of human needs in academia. Marx believed that human needs include material needs and spiritual needs [13]. The American psychologist Maslow believed that human needs include five levels: physiological needs, security needs, social needs, respect needs and self-actualization [14]. Alderfer also simplified them into survival, interaction, and developmental needs [15]. The human demand for ecological land has been expressed in two main areas, these being the demand for products that provide for human life and functions that ensure the quality of human life [16]. Currently, the standard of living for human beings has been gradually improved, and the basic needs of survival have been satisfied, so the demand for ecological land has risen from the two previous demands to a third demand, which is the demand for natural landscape beauty. Therefore, from the perspective of these three levels of human needs, the services provided by ecological land should include abundant and sufficient material products, a healthy and safe ecological environment and a unique and charming landscape culture. The capability of ecological land to provide these products depends on ecological quality and ecological land allocation relationships [17]. Therefore, to meet human needs, it is necessary to optimize ecological land allocation to enhance ecological quality. Ecological land allocation is determined through model calculations and policy guidelines, and implemented through planning [18], and ecological land allocation affects environmental quality and economic development performance [19].

The optimal allocation of land is divided into two parts: quantity optimization and spatial layout optimization. The current academic community mainly employs classical optimization methods such as linear programming, MOP and system dynamics models for land quantity optimization. Steven et al. [20] optimized land use structure in Montgomery County, Maryland using a multi-objective planning model. Sadeghi et al. [21] developed a multi-objective linear programming model for optimal land use allocation in Brimvand watershed, Kermanshahan Province, Iran. Wang, G. et al. [22] established a multi-objective spatial optimization model based on land use classification results to provide land use zoning plans for municipal governments in the context of low-carbon urban development. Termansen et al. [23] proposed a modeling approach for land use dynamics based on choice experiments and spatially explicit system dynamics modeling to predict trends in land use under different environmental and policy scenarios. The study of construction land expansion and the study of optimal allocation of arable land are the main objects for research. 

For spatial layout optimization, scholars mostly apply a cellular automata model (CA) [24], CLUE-S model [25], FLUS model [26], PLUS model [27,28] et al., and hybrid models, such as the CA-Markov model [29], SD-CLUE-S model [30] and SD-CA model [31]. Since the hybrid model integrates the quantitative prediction model and the spatial pattern prediction model, it can optimize the configuration of the quantitative structure and spatial pattern of land use at the same time, and so it is widely used in the study of optimal land use allocation. Each of these models has its own advantages during the simulation and prediction process, but there are also shortcomings. The discrete nature of CA models simplifies many complex problems, and their statistical measures are easy to calculate, making it easy to complete the transition from conceptual models to computer physical models, but the consideration of various macroscopic factors in the simulation process is obviously insufficient. The CLUE-S model has good synthesis, but does not consider enough the influence of natural and policy factors, and overemphasizes the influence of economic benefits. Some model methods also lack the integration of policy, economic and social factors, and are both cumbersome to operate and poorly applied. The CA-Markov model can not only predict the total amount of transfer of various ecological space types and the transfer probability matrix, but it can also simulate the prediction of the spatial distribution of ecological space types through analysis between neighborhoods. However, the relationship between ecological space and its influence factors is very complex and nonlinear, and it is difficult for the CA-Markov model to fully determine the relationship between them to determine the transformation rules.

The patch-generating land use simulation (PLUS) avoids the same shortcomings of the CLUE-S and FLUS models in terms of transformation rule mining and landscape dynamics simulation. It can simulate multiple types of land use changes at the patch level, and the simulation results can be perfectly adapted to multi-objective optimization algorithms [32].

**Figure 1:**
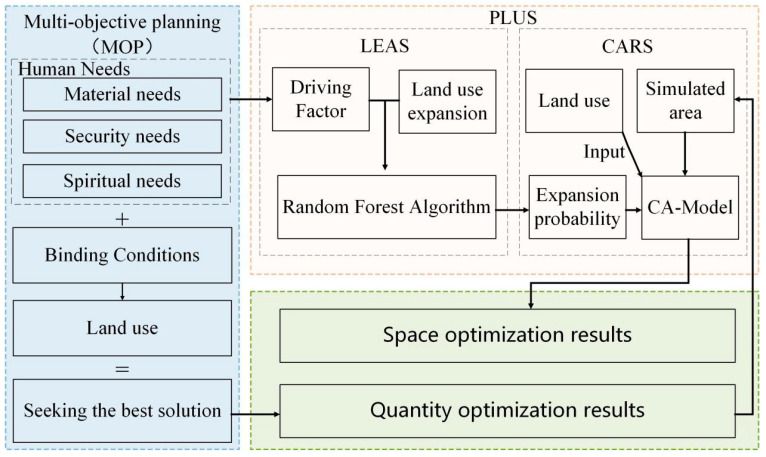
Research idea diagram.

In summary, although there is much research on land quantity and spatial optimization, most of the current studies focus on the optimization of arable land and construction land. These are related to human life through models which ignore the human needs for ecological land and lack the study of ecological land optimization from the perspective of human needs. Hechi City in Guangxi Zhuang Autonomous Region has rich ecological resources, a large proportion of ecological land and relatively backward economic development. The issue of how best to take advantage of the rich natural resources to meet human needs, while enhancing economic growth, is an urgent problem for such areas. Using Hechi City of Guangxi Zhuang Autonomous Region as an example, this paper utilizes the MOP method to measure the optimal amount of ecological land under different human needs objectives at three levels: material needs, security needs and spiritual needs. It then further combines the PLUS model to optimize the spatial layout of ecological land in the study area from a human needs perspective, and proposes an optimal allocation plan for ecological land based on this, which can provide policy guidance for regional land planning. This will provide policy guidelines for regional land use planning, coordination of land use conflicts and the adjustment of ecological red lines. The specific research idea is shown in Figure 1.

## 2. Materials and Methods

### 2.1. Data Sources and Study Area Overview

#### 2.1.1. Data Source

The land use data of Hechi City used in this study were obtained from the 30 × 30 m raster data of the Resource and Environment Science and Data Center of the Chinese Academy of Sciences (http://www.resdc.cn/, accessed on 27 May 2022), and the land use data of 2000, 2005, 2010, 2015 and 2020 were classified as arable land, forest land, grassland, water, residential land and industrial and mining transportation land. The remaining data and specific data sources are shown in Table 1.

#### 2.1.2. Study Area Overview

**Figure 2:**
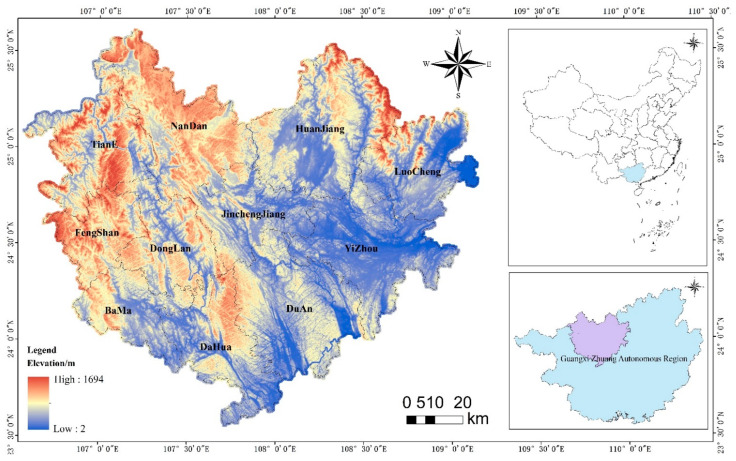
Location map of the study area.

Hechi is located in the northern part of Guangxi Zhuang Autonomous Region (Figure 2), where forest and grass land are the primary land use types. The total area of Hechi is 33,500 km^2^, of which forest land is 24,900 km^2^, accounting for 74.32% of the total area, and grassland is 0.42 million km^2^, accounting for 12.53% of the total area. Hechi covers an area of 0.98 hm^2^ per capita of the resident population, about twice the area per capita of China, and 0.72 hm^2^ of forest land per capita, much larger than the 0.12 hm^2^ of forest land per capita of China. With a large proportion of forested land within Hechi, and a relatively sparse population distribution, the local economic development level is poor. To consider the national key support of the poor areas, it is crucial to explore the amount of ecological land required by the city’s population and the optimal spatial layout to guide the study area in the rational use of ecological land, the expansion of construction land, the use of natural resources and local advantages to improve economic income and people’s life satisfaction.

### 2.2. Research Methodology

#### 2.2.1. Multi-Objective Planning Model

The basic expression of the MOP model is:

(1) $$f\left(x\right)=\sum_{j=1}^{n}c_{j}x_{j}$$

The constraints are:

(2) $$\sum_{j=1}^{n}a_{\mathrm{ij}}X_{j}=\left(\geq ,\leq \right)b_{i}\;i=1,2,\;\ldots \;,m$$

where f(x) is the objective function; X_j_ is the area of various types of land; c_j_ is the coefficient; a_ij_ is the constraint coefficient; b_i_ is the constraint constant; m is the order, indicating the number of constraints; n is the dimension, indicating the number of decision variables.

**Figure 3:**
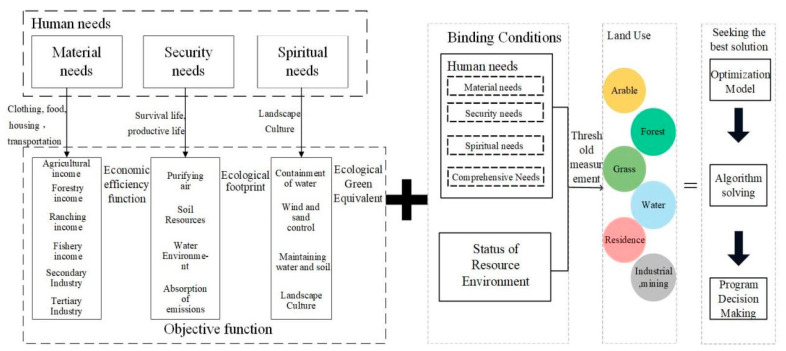
Multi-objective planning flow chart.

Combining the current situation of ecological land in Hechi and the characteristics of land use, the variables were determined to be arable land (X_1_), forest land (X_2_), grassland (X_3_), water (X_4_), residential land (X_5_) and industrial and mining transportation land (X_6_), a total of six variables. In this paper, we first set up a non-disturbance linear development scenario (LD) for the evolution of land use in Hechi City. In addition, we set up four scenarios using the MOP method, namely, the priority human security needs scenario, the priority human spiritual needs scenario, the priority human material needs scenario and the comprehensive human needs scenario. The amount of ecological land to meet human needs in 2035 was achieved by projecting the land use needs under different scenarios. The specific research process of MOP is shown in Figure 3.

(1) LD Scenario. Based on the trend of each land use land type from 2000 to 2020, the amount of ecological land in Hechi City in 2035 was linearly projected. This scenario assumes that each locality is not disturbed by external factors and only develops linearly according to its natural development trend, which is a land use change scenario with no human intervention.

(2) Prioritize human security needs scenarios. In this scenario, only human security needs are considered, and the ability of ecosystems to influence temperature and precipitation, absorb greenhouse gases and provide clean air and a climate suitable for human survival. The ecological footprint measures the impact of human consumption on nature by tracking the pressure of human needs in terms of arable land (area needed for food), grassland (area needed for livestock), forests (for paper and timber production), building land (area needed for infrastructure and housing), carbon dioxide footprint (forests needed to absorb carbon dioxide) and watersheds (needed to produce aquatic products) [33]. Security needs are similar to the ecological footprint, so this paper uses the ecological footprint to represent human security needs.

The ecological footprint model includes two parts: the ecological footprint and the ecological carrying capacity. The ecological footprint refers to the biologically productive geographical area that can accommodate the waste emitted by humans [34]. This paper uses ecological footprint data for the measurement of human security needs, and is calculated as follows:

(3) EF=N×ef

(4) $$\mathrm{ef}=\sum_{j=1}^{4}\left[\sum_{i=1}^{n}r_{j}\times \frac{c_{i}}{p_{i}}\right]$$

where: EF for Ecological Footprint (hm^2^); N is the total population of the region; ef is the ecological footprint per capita (hm^2^·cap^−1^); j is the land type; r_j_ is the equilibrium factor; n is the number of consumer product types; i is the type of consumer item; c_i_ is the annual per capita consumption of the *i*th consumption item (kg·cap^−1^); p_i_ is the national average production capacity of the *i*th consumption item (kg·hm^−2^). The equilibrium factor was measured by Liu Moucheng and Li Wenhua [35,36] for China. Each province, city and the specific indicators are shown in Table 2. The calculation leads to:

(5) $$f_{1}\left(x\right)=0.02x_{1}+0.08x_{2}+0.45x_{3}+0.16x_{4}$$

Therefore, the expression of the multi-objective optimization function for the scenario of prioritizing human security needs is Max f_1_(x).

(3) Prioritizing human spiritual needs scenarios. Spiritual needs refer to people’s needs for natural landscape beauty, culture, etc. In this scenario, we consider more the landscape recreation provided by ecological sites, which provide opportunities for human observation, research and understanding of ecosystems, or the ability to serve as objects for scientific research and education. According to the existing research results [37], ecological green equivalents are applied to define human spiritual needs. The ecological green equivalent is defined as the ratio of the amount of green of different green plants to the amount of green of the same amount of forest area. It can be understood that green vegetation produces photosynthesis, whether it is arable land such as paddy field, dry land, watered land, garden land such as orchards, tea plantation, rubber plantation, or grassland such as natural pasture or artificial pasture, it produces the same amount of ecological function equivalent to that of forest. The ecological green equivalence is proposed mainly in consideration of the fact that natural green plants other than forests can, to a certain extent, play an ecological compensatory role in the environment and serve as a landscape function for human beings. The objective function of human spiritual needs is:

(6) $$f_{2}\;\left(x\right)\;=\left(\frac{\sum_{j=1}^{7}g_{i}x_{j}}{S}\right)\times 100\%$$

g_i_ is the average green equivalent of each type of land, according to the relevant literature, and the calculation of g_i_ is mainly based on the scoring table of various ecological functions obtained by Japanese experts according to the survey method [38]. The ecological green equivalents were obtained by combining the results of related scholars, and on this basis, the ecological green equivalents were obtained based on the growth period and maturation system. The crops in Hechi are biannual, so we need to multiply the growth period coefficient by 0.67, and finally obtain the average green equivalent g_i_, g_1_ = 0.33, g_2_ = 1, g_3_ = 0.34, g_4_ = 0.83. In contrast, residential land, industrial and mining transportation land and unused land do not provide ecological landscape function services for human beings, so there is no ecological green equivalent, so, g_5_ = g_6_ = g_7_= 0. S is the total land use area of the study area. Equation (6) can be rewritten as:

(7) $$f_{2}\;\left(x\right)\;=\left(\frac{0.33x_{1}+x_{2}+0.34x_{3}+0.83x_{4}}{S}\right)\times 100\%$$

Therefore, the expression of the multi-objective optimization function for the scenario of prioritizing human spiritual needs is Max $f_{2}\left(x\right)$.

(4) Prioritization of human material needs scenarios. In this scenario, human material needs are prioritized. Human material needs refer to people’s needs for clothing, food, housing, transportation and other related goods, labor tools, cultural goods, etc., which can be divided into needs for means of living and means of production. Based on the actual situation of the region, each land use type is fully utilized to maximize economic benefits. This function can help grasp the risk of land use conflicts in the developing city of Hechi, which prioritzes the satisfaction of human material needs. It reveals the potential threat of the GDP-oriented development model to social stability and environmental carrying capacity. The formula for estimating economic benefits is as follows:

(8) $$f_{3}\left(x\right)=\sum_{i=1}^{7}E_{i}\times x_{i}$$

where f_3_(x) is the total economic benefit, E_i_ is the economic benefit of the ith land use type per unit area (RMB/km^2^); x_i_ refers to the area of the *i*-th land use type (km^2^), referring to existing studies [27,39] to estimate the economic benefits of cropland, woodland, grassland and watershed from agriculture, forestry, livestock and fisheries, respectively. Estimation of economic benefits of industrial, mining and transportation land use and residential land use using the gross output value of secondary and tertiary industries. Based on the statistical yearbooks of 2000, 2005, 2010, 2015 and 2020 and the land use data, the gray prediction model GM (1,1) is used to estimate E_i_, then Equation (8) can be rewritten as:

(9) $${\mathrm{maxf}}_{3}\left(x\right)=362.33x_{1}+13.56x_{2}+168.47x_{3}+235.78x_{4}+45783.88x_{5}+67582.55x_{6}$$

Therefore, the expression of the multi-objective optimization function for the scenario of prioritizing human security needs is Max $f_{3}\left(x\right)$.

(5) Comprehensive human needs scenarios. Human needs always need to be satisfied simultaneously, and the three needs can promote each other and complement each other. This scenario is important for integrating human needs, promoting the development of Hechi City in meeting all human needs, and promoting the efficient development and utilization of national land resources. The multi-objective optimization function in this scenario is expressed as:

(10) $$\mathrm{Max}\left\{f_{1}\left(x\right),f_{2}\left(x\right),f_{3}\left(x\right)\right\}$$

The objective function under each human need will be constrained by realistic conditions; based on the existing territorial spatial planning and related research, the constraints of multi-objective planning model are developed (Table 3). The established objective functions and constraints are imported into MATLAB R2017b to model, solve, and obtain the land use needs under different human needs objectives. Together with the land use needs under LD scenarios, the land use pattern under five scenarios is simulated in the PLUS model, and the constraints of multi-objective planning are shown in Table 3.

#### 2.2.2. The Patch-Generating Land Use Simulation (PLUS)

The PLUS model contains two parts, LEAS and CARS, and this study mainly uses the PLUS model to calculate the land use pattern of Hechi City in Guangxi, under multiple scenarios, to realize the analysis of land use change against the goal of human demand. Additionally, it uses the extended land use analysis strategy (LEAS) in the PLUS model to analyze the influence of human demand on ecological land change. The first step of the PLUS model is LEAS. The strategy works by extracting the changes of each type of land between different time phases. A random forest algorithm was used to explore the factors and drivers of various types of land use expansion from a sample of new sections. The calculation formula is:

(11) $$C_{i,k}^{d}\left(x\right)=\frac{\sum_{n=1}^{M}I\left[h_{n}\left(x\right)=d\right]}{M}$$

where: x is a vector composed of multiple drivers; h_n_(x) is the predicted type of the nth decision tree of vector x; the value of d is 0 or 1, where 1 indicates the presence of other land use types transformed into this k class of land use types and 0 indicates other transformations; M is the total number of decision trees and I is the indicator function of the set of decision trees.

The second step of the PLUS model is CARS. Combining stochastic seed generation and decreasing threshold mechanism, the PLUS model can simulate the patch generation dynamically in time and space under the constraint of development probability. Through this step, the ecological land layout of the study area in 2035 based on human needs perspective is simulated. The module contains two main parts.

(1) The feedback mechanism of macro demand and local competition, which mainly reaches the generation of multi-class random patch seeds, and then simulates the calculation of the overall probability of land use type k, The calculation formula is:

(12) $${\mathrm{OP}}_{i,k}^{d=1,t}=P_{i,k}^{d=1}\Omega_{i,k}^{t}D_{k}^{t}$$

where P_i,k_^d=1^ represents the growth probability of land use type k of raster i; D_k_^t^ is the effect of future demand for land use type k, an adaptive driving coefficient that depends on the gap between the amount of land use and demand for land use type k in period t from the iteration; Ω_i,k_^t^ represents the neighborhood effect of raster i, i.e., the proportion of land use type k that is covered in the next neighborhood. CARS constructs a roulette wheel based on the overall probability of all land use types, which is used to select the land use status in the next iteration.

(2) Decreasing seed threshold for multi-class stochastic patches. The PLUS model evolves patches of multiple land use types by calculating the overall probability of utilization type process through the decreasing seed threshold trend of multi-class stochastic patches, and when the neighborhood effect of land use k is equal to 0, the overall probability is:

(13) $${\mathrm{OP}}_{i,k}^{d=1,t}=\left\{\begin{array}{c} P_{i,k}^{d=1}\left({r\mu }_{k}\right)D_{k}^{t}\;\mathrm{When}\;\Omega_{i,k}^{t}=0\;\mathrm{and}\;r<P_{i,k}^{d=1}\\ P_{i,k}^{d=1}\Omega_{i,k}^{t}D_{k}^{t}\;\mathrm{Other} \end{array}\right.$$

where r is a random value ranging from 0 to 1 and is the threshold value for newly generated land use type k patches. The seeds generated in the PLUS model can grow into new land use type grids and gradually form new patch groups. The decreasing threshold rule adopted by the PLUS model in the process of site type competition uses the threshold τ to evaluate the land types identified by roulette to limit the spontaneous growth of land types and drive better results.

The influence factors refer to the study setting of Liang et al. [32]. The accuracy of the model was verified using land use data for 2010 and 2020, and the kappa coefficient was more significant than 0.85, meeting the study requirements. The influence factors are set as follows: material needs influence factor, security needs influence factor, spiritual needs influence factor, and the specific factor expression is as follows (Table 4):

(1) DEM: the impact of DEM on land use change is pronounced in areas with rugged terrain and large differences in posters, and the change of DEM is also obvious in areas with more mountainous and forested landscapes in the study area, which can meet the ecological security needs and spiritual needs of human beings.

(2) Slope: the gentler the slope, the more likely land use change will occur [40], land use change from ecological to built-up land is more likely to occur on land with lower slopes [41]. Areas with low slopes are more likely to produce products for human material needs and to provide natural landscapes for human spiritual needs.

(3) Distance to railroads, distance to roads: accessibility is a crucial factor influencing land use change. Good accessibility is a driving force for investment, accelerates the construction of urban infrastructure, attracts agricultural and industrial enterprises, and has an important impact on the transportation of products from ecological lands. Thus, accessibility is an essential factor for land use to meet human material needs.

(4) Population density: population density is expressed by the kernel density of point of interest (POI), which refers to where human beings live. The closer the ecological patch is to the point of interest, the more frequently human beings use the ecological patch and the stronger the needs, i.e., the needs intensity of the ecological patch is proportional to the number of people served. Therefore, high needs intensity is often accompanied by high material and security needs.

(5) GDP: GDP is the final result of the productive activity of all resident units in a country or region during a certain period of time. It is an intuitive representation of the ability of the land to supply the material needs of human beings.

(6) Distance from the river: this can characterize the human needs for natural landscape diversity, and areas closer to the river can provide more landscape cultural services in terms of recreation and aesthetic enjoyment of the landscape.

## 3. Results

### 3.1. Multi-Scenario Simulation of Ecological Land Quantity Optimization

Land use needs under different human needs objectives were obtained by modeling solutions through MATLAB R2017b, as shown in Table 5. Under the security needs objective, compared with the other objectives, the largest area of forest land is 3919.27 km^2^. Relative to the status quo in 2020, only forest land and grassland interconversion, the change area is 288.62 km^2^. Relative to the LD scenario, arable land and forest land increased by 32.44 km^2^ and 308.46 km^2^, respectively. Under the material needs target, the area of arable land is the largest, at 4705.12 km^2^. Except for forest land, all sites have increased relative to the status quo in 2020, with the most noticeable seen in arable land, at 785.85 km^2^. Compared to the LD scenario, the area of arable land, grassland and water has increased, while forest land has decreased by 1143.78 km^2^, and the area of construction land is basically the same. Comparing the spiritual needs target with the current situation in 2020, it can be concluded that only forest land, grassland and water area have changed, among which forest land has decreased by 902.01 km^2^, grass land has increased by 839.66 km^2^, and water area has increased by 62.23 km^2^. Compared with the LD scenario, water area, grass land and arable land have increased, while forest land, residential land and industrial and mining transportation land have decreased. Under the comprehensive human needs target, the area of forest land decreased by 1698.35 km^2^ relative to the status quo in 2020. Except for forest land, where the area of arable land is similar to the material needs target, grassland and water area are similar to the spiritual needs target, and residential land and industrial, mining and transportation land combine the changes under the three targets. Compared to the LD scenario, the arable land significantly increased. The change in the LD scenario shows that without disturbance, the area of arable land will gradually decrease, and residential land and industrial and mining transportation land will blindly increase and occupy arable land.

It can be concluded that under the objective of human needs, the expansion of land for construction is limited, and there is a certain increase in arable land and water area. The significant increase of arable land is because it meets the needs of material creation, ecological safety and landscape pattern at the same time, and arable land is more inclined to material output. Grass land also meets the needs of material output, ecological safety and landscape pattern, but grass land is more expressed in animal output, which is indirect output, and in landscape pattern, so there is a large increase in meeting spiritual needs. The current amount of ecological land in Hechi is unreasonable because there is less arable land, which cannot fully guarantee the material needs of human beings. There is more forested land and, except for the goal of meeting human security needs, the amount of forested land for the rest of the goals is reduced compared to the current forested land, which can be properly developed. If human needs are not considered, according to the natural development trend, the amount of arable land in Hechi will be further reduced. The area of forest and grassland will also be reduced, the area of water will be increased and the amount of residential land and industrial and mining transportation land will be increased.

### 3.2. Multi-Scenario Simulation for Spatial Optimization of Ecological Land

#### 3.2.1. Analysis of LEAS Results

**Figure 4:**
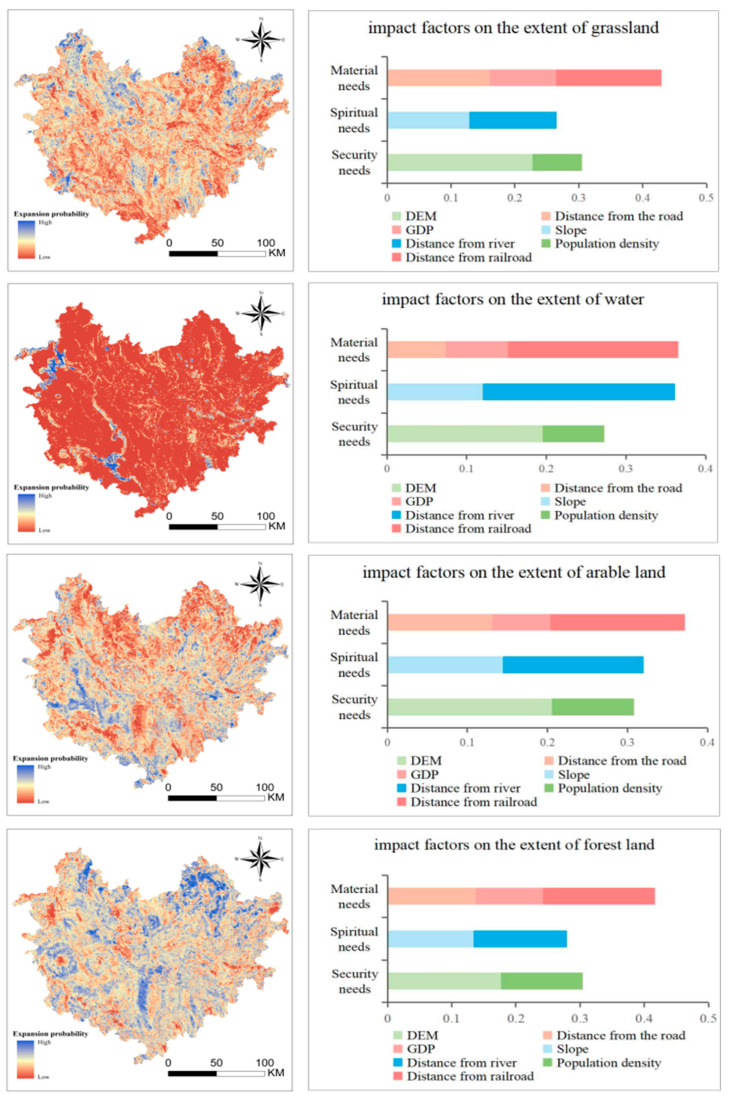
The degree of influence of each influence factor on different land use types and the probability of land use expansion.

The LEAS module of the PLUS model was used to calculate the expansion probability of various types of sites and to investigate the impact of human needs on land use, the reliability of the driver contribution is described by α. Finally, the contribution of each factor to the land expansion is found (α = 0.743, RMSE = 0.133). The larger the value, the greater the influence of the factor on the change of the category, and the results are shown in the figure below (Figure 4).

Overall, it seems that human material needs have the greatest impact on ecological land use, because human material needs are basic needs, and it is only after they are satisfied that humans attend to their other needs. Spiritual needs are secondary to material needs for watersheds and cropland, and security needs are secondary to woodlands and grasslands. Forests and grasslands mainly provide carbon sequestration and oxygen release, and provide a healthy and safe ecological environment for human beings, so security needs have a greater impact on forests and grasslands. Cultivated lands and waters provide production materials and also provide places for human recreation and fun, so human spiritual needs have a greater impact on cultivated lands and waters.

By type of land, regarding the change of arable land, the influence factor values of material needs, spiritual needs, and security needs are 0.37, 0.32, and 0.3, respectively. Distance from railroads accounts for the largest proportion of material needs, followed by distance from roads with 0.16 and 0.13, respectively, and distance from rivers and slope with 0.17 and 0.14, respectively, for spiritual needs. Through the map of arable land expansion probability, it can be understood that the high expansion probability of cropland is distributed in the southwestern region, and the expansion probability is lower in the northern and southern regions. The values of the influence factors of material needs, security needs and spiritual needs for forest land are 0.41, 0.3, and 0.28, respectively. The DEM accounts for a larger share of security needs with a value of 0.17. Forest land has a higher probability of expansion in the northern and southern regions and shows overall ease of change, with fewer areas of low probability and scattered distribution in the western region. For grassland change, the influence factor values of material demand, security demand, and spiritual demand are 0.43, 0.3, and 0.26, respectively. Among security demand, DEM data has the greatest influence on grassland at 0.23, and since a large number of grasslands are in plain areas, these gentler sloping areas have a higher the probability of grassland shifting, which is because grassland in flat areas is easier to develop. The probability of grassland expansion generally shows low change. The probability of grassland expansion generally showed low variation level, with high variation probability areas distributed in the central and northern regions. For watersheds, the influence factor values of material needs, spiritual needs and security needs are 0.36, 0.36 and 0.27, respectively. The influence of spiritual needs and material needs on the watershed is equally high because watershed not only has rich material output but also provides resting places for human beings. The overall probability of change in watershed shows a low level because watershed cannot be expanded at will, and compared with woodland, grassland, forest land and cropland, watersheds have the highest requirements for human land use activities and surface environmental reshaping.

#### 3.2.2. Results of Spatial Optimization of Ecological Land in Hechi

**Figure 5:**
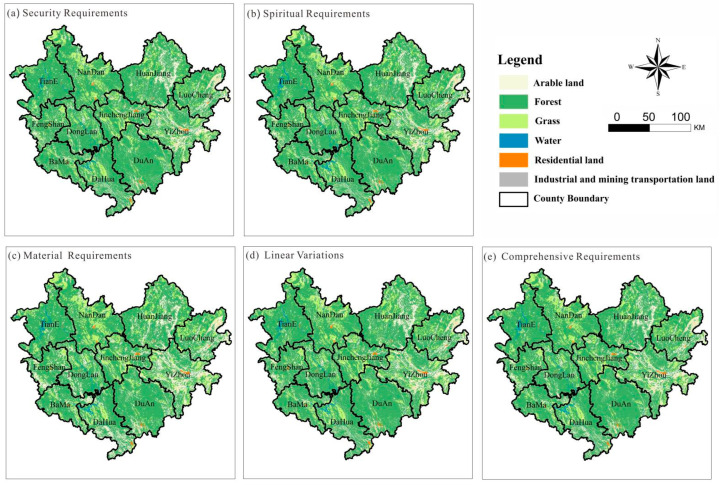
Land use map of Hechi City in 2035 under meeting different human needs goals. (**a**) Security requirements optimization results, (**b**) Spiritual Requirements optimization results, (**c**) Material requirements optimization results, (**d**) Linear variations optimization results, (**e**) Comprehensive Requirements optimization results.

The land use pattern under five scenarios was simulated using the land use structure derived from MOP to meet different human needs in the PLUS model and combining it with the land transfer probabilities calculated from LEAS. In the spatially optimized transfer rule, referred to in the existing research [27], the probability of transferring residential land and industrial, mining and transportation land is set to 0 because construction land is not easy to change. The area within the red line of arable land protection, in the result of “three zones and three lines” of Hechi City, is taken as the no transfer area, and the parameters in the model are set as follows [32]: the patch generation threshold is set to 0.5, the higher the value, the less likely the site type is to be converted; expansion coefficient is set to 0.1, denotes the probability of random plaque seeding, ranging from 0 to 1, with higher values indicating more likely to generate new plaques. The resulting results are shown in the figure below (Figure 5):

Figure 5a, in the northern part of Hechi, Tian’e County, Nandan County, Huanjiang Maonan Autonomous County and Luocheng Mulao Autonomous County, under the goal of security needs, the woodlands significantly increased. They displayed a trend of agglomeration, and the connectivity of forests was improved.

Figure 5b, under the material needs target, the arable land in the southwestern region as well as the eastern region, Fengshan County, Donglan County and Yizhou City, significantly increased, while the arable land in the northern region, Huanjiang Maonan Autonomous County, was heavily occupied by forest land. The topography of Huanjiang is highly undulating, so it is difficult to implement land leveling projects and cultivate. While the eastern and southwestern areas are relatively gentle sloping, so arable land has increased a lot in this part of the country, while large amount of forest land in the eastern area has been transformed into grassland with the same factor. Therefore, the land for construction is expanded appropriately according to the population, which also meets the human needs for ecological land.

Figure 5c, under the spiritual needs objective, similar to the change in security needs, there is a significant increase in forested land in the northern region. It is worth noting that the area of water in the northwest region significantly increases. The area is Longtan Reservoir, with a natural landscape tourism area and convenient transportation to meet the human needs for natural landscape. Residential land and industrial and mining traffic are almost unchanged.

Under the comprehensive needs target shown in Figure 5d, the overall land use has become more connected, less fragmented and more concentrated areas of forest, arable land and grassland. There is a significant increase in forest land in the northeast and a significant increase in arable land in the southwest, and a significant widening of the Hongshui River basin, which runs almost north and south of Hechi and provides rich fishing resources, ecological landscapes and human resting places in its coastal areas. The residential land and industrial, mining and transportation land remain unchanged, and the uncontrolled expansion of construction land is restrained.

The above results suggest that the study area should be restrained from the disorderly expansion of construction land, the ecological land gathering should be improved, the fragmentation of arable land, forest land and grassland should be reduced, and the southwestern region should be concentrated in continuous cultivation to ensure crop yield. In the global context of the COVID-19 epidemic, we are always alert to the problem of “food security”, and we should give full play to the natural background of the southwestern region to cultivate relatively gentle sloping areas to reduce the problem of arable land abandonment, to meet human material needs and to improve the local economic development in the form of crop output. Longtan Reservoir and the coast of the Hongshui River basin should develop the advantages of the natural landscape of mountains and water, actively protect ecological land and improve the quality of the water environment and water ecology. The northeastern region develops a large number of forest industries, makes full use of woodland resources, scientifically develops forest food, forest grass, forest mushroom, forest medicine intercropping and forest poultry, forest livestock composite operation and realizes the short term to increase the long term, improving the comprehensive benefits of forestry, feeding people with forest, enriching people with forest.

### 3.3. Results of Internal Optimization of Ecological Red Lines

**Figure 6:**
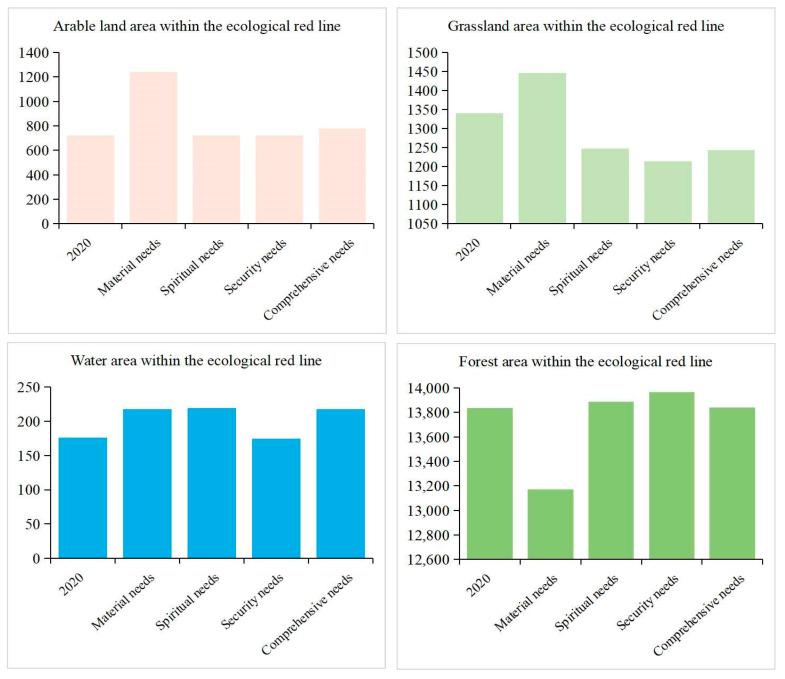
Amount of ecological land inside the ecological red line after different optimization results.

The delineation of ecological red lines is a conservation measure to meet human needs in China. The resulting optimization results intersected with the ecological red line of Hechi City, resulting in the optimized ecological land within the red line, as shown in Figure 6.

It can be seen that forest land still dominates within the ecological red line, followed by grassland and arable land, and the area of water is the smallest. In general, the area of arable land within the ecological red line is the largest under the material demand scenario, with 1242.06 km^2^. The area of forest land is the largest in security needs scenario, with 13,967.11 km^2^. The area of grassland is the largest under the material needs scenario, with 1445.55 km^2^. The area of the watershed is the largest under both the integrated human demand scenario and the material demand scenario, with 217.92 km^2^.

In each scenario, under the material demand scenario, arable land, grassland and water area all increase compared to 2020, with arable land increasing the most, at 520.39 km^2^, while forest land decreases, at 667.10 km^2^. Under the spiritual demand scenario, the arable land is basically the same as the area within the red line in 2020. Forest land and water area are increased, by 52.28 km^2^ and 42.93 km^2^, respectively, and grass area is decreased by 93.36 km^2^. Under the security demand scenario, the area of arable land and water area decreases less than that in 2020, by 1.13 km^2^ and 1.63 km^2^, respectively. Forest land increases by 128.93 km^2^, and grass area decreases by 125.42 km^2^. Under the comprehensive needs scenario, the area of arable land and water within the red line increases by 55.04 km^2^ and 41.50 km^2^, respectively, compared with 2020. Forest land remains almost the same, and grassland decreases by 96.86 km^2^.

Under the material needs, a large increase in the area of arable land likewise leads to a significant increase in arable land within the ecological red line. In the process of increasing arable land, a large area of forest land is inevitably occupied, thus leading to a decrease in the area of forest land within the ecological red line. Under the spiritual needs, the increase of forest land and water area within the red line can further enhance the ecological supply capacity provided by the red line. Under the security needs, the transformation between woodland and grassland within the red line is similar to the overall change trend. The slight increase in arable land and waters within the ecological red line and the slight decrease in grassland under the comprehensive human needs scenario are also similar to the overall change, increasing the direct supply capacity of ecological land. Except for the material needs scenario, the ecological land within the ecological red line does not change much, which indicates that the current ecological land ratio within the ecological red line is satisfactory, and only a small amount of land has to be adjusted to meet human needs within the ecological red line. In contrast, the material needs scenario starts from maximizing economic benefits, so there is a large increase in arable land and a large decrease in forest land, and so the ecological red line needs to be extensively adjusted under this demand target. The ecological red line needs to be adjusted on a large scale.

## 4. Discussion

### 4.1. It Is Scientifically Significant to Combine the MOP-PLUS Model from the Perspective of Human Needs

Land is a critical resource of limited and unevenly distributed national space, national spatial governance focuses on the allocation of land resources to achieve efficient, equitable and sustainable utilization of land resources by meeting the land needs of different human demands and coordinating the interests of all parties [42], which is highly compatible with the scenario design concept of this study. Therefore, the MOP-PLUS method is an important method by which to seek the harmonious development of the human-land relationship, and making decisions on the amount of land use at different levels of human needs, constructing spatial governance programs from a “human-centered” perspective, establishing a “top-down” spatial governance system, promoting the “integration of multiple regulations” and improving the national spatial planning system and national spatial control programs [43].

Fewer studies have been conducted in the field of land in conjunction with human needs. For the study of human needs, literature mainly focuses on human needs in addressing socio-economic and ethical issues such as poverty, social well-being, social exclusion and human rights. The study of human needs is abundant in the fields of philosophy (from early Greek philosophers to Marx, Rawls or Habermas), sociology [44], psychology [14], religious studies [45] and economics [46]. Human needs are interpreted differently in different fields, and there is no consensus in the academic community about the exact meaning of human needs. In general, the satisfaction of human needs is defined subjectively as the satisfaction of perceived well-being and desires, or objectively as the satisfaction of the quantity of necessities. The use of ecological land is closely related to human needs [47]. In the field of land use research, human needs and ecological land are rarely combined. By analyzing the amount of various types of land in the region from the perspective of human needs, we can scientifically plan local development and formulate ecological land use planning for the region from the perspective of human needs, drawing on the experience of typical regions.

### 4.2. The Reduction of Forest Land in the Study Results Needs to Be Considered with Caution

Compared with other scholars’ studies, Duan et al. [48] used MOP-PLUS to optimize the simulation of the Songhua River basin in northeastern China, and the final results yielded a decrease in forested land under the economic development scenario, and the results were similar to this paper. However, under the water conservation scenario and the ecological conservation scenario, the forest land increased due to the consideration of functions such as water conservation and biodiversity protection of forest land. Similarly to the study area of this paper, the main land use type of the Songhua River basin is forest land, and both are areas where economic development and land resource development are severely restricted. However, the starting point of this paper is the protection of the natural environment, while the starting point of this paper is the satisfaction of human needs, so the results are different.

However, from the ecological point of view, forest areas are the main area for biodiversity conservation, have high carbon absorption capacity, and are of supra-regional importance. We believe that the reduction of forest areas in the results of this paper is scientifically reasonable within the study area, although the high value area of resource and environmental carrying capacity evaluation is taken as the prohibited conversion area in the spatial simulation of this paper, considering the high quality forest areas that are not allowed to be transferred. However, the reduction of forest land must be considered alongside overall human interest, which is the direction of our next research study, and expands the scope of this study to discuss the changing trend of globalized land to forest land.

### 4.3. The Land Can Be Used More Efficiently after Considering Human Needs

Under the objective of considering human needs, the expansion of land for construction is limited, and there is a certain increase in arable land and water area. The large increase in arable land is because arable land meets the needs of material output, ecological safety and landscape pattern at the same time, and arable land is more inclined to material output. However, grassland is more relevant to animal output, which is indirect output, and is more inclined to landscape pattern, so there is a significant increase under the goal of meeting spiritual needs. If human needs are not considered, according to the natural development trend, the amount of arable land in Hechi City will be further reduced in the future. The area of woodland and grassland will be similarly reduced, and the water area will be increased along with residential land and industrial and mining transportation land.

### 4.4. The Study Area Should Adjust the Ecological Land Structure and Enhance the Ecological Land Aggregation According to the Research Results

The results of this paper show that the ecological land in the study area, after considering the human demand perspective, is still dominated by forest land, with an appropriate increase in arable land and watershed and a decrease in grassland. In terms of spatial layout, there is a change of concentrated contiguity in forest land in the northeast and arable land in the southwest. The study area should enhance the aggregation of ecological land and reduce the emergence of fragmented arable land, forest land and grassland, while restraining the disorderly expansion of construction land, and the southwestern region should concentrate continuous farming to ensure crop output. In the global context of the COVID-19 epidemic, we are always alert to the problem of “food security”. We should give full play to the advantages of the natural background of the southwestern region, cultivate in relatively gentle slope areas to reduce the problem of arable land abandonment, meet the material needs of human beings and improve the local economic development in the form of crop output. Longtan Reservoir and the coast of the Hongshui River basin should develop the advantages of the natural landscape of mountains and water, actively protect ecological land and improve the quality of the water environment and water ecology. The northeastern region develops a large number of forest industries, makes full use of woodland resources, scientifically develops forest food, forest grass, forest mushroom, forest medicine intercropping, forest poultry, forest livestock composite operations, realizes the short term to advance the long term, improves the comprehensive benefits of forestry, feeds people with forest, enriches people with forest.

This paper uses the MOP-PLUS model, combined with human demand, to derive the land use structure and spatial layout of Hechi City under different scenarios. The results are more accurate, with errors occurring within acceptable limits, and provide a research direction for predicting land use demand based on the subjective human perspective. It is also shown that the model solves the problem of land allocation in urban land use under the perspective of meeting human needs. This method can provide technical support for the formulation of future territorial spatial planning in China and can be applied to cities with more extensive ecological land use to guide their development direction. In this study, the land use classification should further differentiate the land use types in order to more accurately guide the future land use development in the study area. In the land use optimization simulation, the problem of model accuracy needs to be further considered to improve the credibility of the prediction results, and the PLUS model can be further optimized by combining intelligent body models such as particle swarm algorithm and ant colony algorithm. Due to the specificity of policy factors, we cannot reasonably digitize and vectorize them for the time being, so the impact of policy factors is not discussed in the discussion of drivers, which is something we need to consider in our next study.

## 5. Conclusions

In this paper, we use the MOP-PLUS model using Hechi, Guangxi as the study area. The impact of human needs on the change of ecological land use was investigated from four perspectives: material needs, security needs, spiritual needs and common satisfaction, and the demand for ecological land and its layout in 2035 was simulated and predicted.

(1) In terms of quantity, under the objective of human needs, the expansion of land for construction is limited, but there is a certain increase in arable land and water, and the large increase in arable land is due to the fact that arable land meets the needs of material creation, ecological security and landscape pattern at the same time, and arable land is more inclined to material output. Grassland also meets the needs of material creation and ecological security, but grassland is more expressed in animal output, which is indirect output, and is more inclined to landscape pattern, so there is a large increase in meeting the spiritual needs.

(2) In terms of land use layout, the future development of Hechi should restrain the disorderly expansion of construction land from meeting human needs, enhancing the gathering of ecological land, reducing the emergence of fragmented arable land, forest land and grassland, and concentrate continuous farming in the southwestern region to ensure crop output.

(3) In the optimization of the ecological red line, except for the material demand scenario, the ecological land within the ecological red line does not change much, indicating that the current ecological land ratio within the ecological red line is good. Only a small amount of land needs to be adjusted to meet the human demand within the ecological red line.

(4) If the development is carried out according to the laws of nature without considering human needs, it will inevitably lead to the expansion of construction land to occupy ecological land, and eventually ecological land cannot meet the basic human needs.

## Figures and Tables

**Table 1:** Data source table.

Name	Source
DEM	Geospatial Data Cloud (https://www.gscloud.cn/, accessed on 29 May 2022) 30 × 30 m raster data
Slope	Calculated using DEM data
Land Use Data	Resource and Environmental Science and Data Center, Chinese Academy of Sciences (http://www.resdc.cn/, accessed on 27 May 2022) 30 × 30 m Raster Data
Population, land constraints	«Hechi City Territorial Spatial Master Plan (2021–2035)»
Population density	https://www.worldpop.org, accessed on 29 May 2022, 1 km × 1 km Raster Data
GDP	Resource and Environmental Science and Data Center, Chinese Academy of Sciences (https://www.resdc.cn, accessed on 29 May 2022) 1 km × 1 km Raster Data
Economic Data	«Hechi City Statistical Yearbook» (2000–2020)
Vector data of Ecological red line, rivers, roads, etc.	«Hechi City Territorial Spatial Master Plan (2021–2035)» Database

**Table 2:** Ecological footprint model indicator evaluation system.

Type of Product Consumption	Land Use Type	Consumption Items	Ecological Footprint Balance Factor
Bioresource consumption	Arable Land	Rice, wheat, corn, sorghum, cereals, beans, sweet potatoes, peanuts, rape, sesame, cotton, hemp, sugar cane, tobacco, cassava, other crops, pork, poultry, poultry eggs	1.74
	Grassland	Beef and lamb, other meat, wool, milk, rabbit fur	0.44
	Forest Land	Oil tea seeds, pine resin, walnuts, chestnuts, wood, bamboo, other fruits	1.41
	Water Aera	Fish, crab, shellfish, other freshwater products	0.35

**Table 3:** Constraint description table.

Formula	Description
$\sum_{i=1}^{6}x_{i}=$ 33,481.23	1. Total area constraint: With reference to the Hechi City Territorial Spatial Master Plan, the total area of each land use type is set at 33,481.23 km^2^ and remains unchanged.
4,523,100 ≤ 120 (x_1_ + x_2_ + x_3_) + 3000 (x_5_ + x_6_) ≤ 4,618,000	2. Total population constraint: Based on the historical population statistics of the study area and with reference to the Hechi City Territorial Spatial Master Plan, the population of the study area in 2035 is the maximum number of people under the high development scenario constraint and the minimum number of people under the low scenario, where the population density of arable land, forest land and grassland is 120 people/km^2^, and the construction land is 3000 people/km^2^.
*x*_1_ ≥ 3919.27	3. Food security constraint: Based on the food security perspective, the total arable land area in the study area is not allowed to decrease relative to the area in 2020 based on the Hechi City Territorial Spatial Master Plan.
136.49 ≤ *x*_5_ ≤ 143.27	4. Construction land constraint: Considering the construction land is not easy to change, with reference to the “Hechi City Territorial Spatial Master Plan”, the high development plan for construction land is the upper limit, and the current construction land area is the lower limit.
*x*_4_ ≥ 311.17	5. Water Bodies Constraint: The area of water bodies in Hechi City is increasing yearly, and the ecological value of water bodies is significant, so the area of each water body should be at least not less than the area in 2020.
32,787.82 ≥ *x*_1_ + *x*_2_ + *x*_3_ + *x*_4_ ≥ 32,527.23	6. Ecological footprint constraint: A gray prediction model GM (1,1) was used to obtain the ecological footprint area per capita in the study area in 2035, and then multiplied by the population in 2035, with the population at the high development scale as the upper limit and the population at low development scale as the lower limit to obtain the ecological footprint constraint.
*x_i_* ≥ 0 (I = 1, 2…,7)	7. Mathematical model constraint: all types of variables cannot have negative values.

**Table 4:** Table of impact factor types.

Impact Factor	Form of Representation
Security needs	DEM
	Population density
Spiritual needs	Distance from river
	Slope
Material needs	Distance from railroad
	Distance from road
	GDP
	Population density
	Slope

**Table 5:** Quantity optimization with multiple human needs goals (Unit (km^2^)).

Type of Land Use	Area in 2020	Security Needs Objectives	Material Needs Objectives	Spiritual Needs Objectives	Comprehensive Needs Objectives	LD Scenario
Arable land	3919.27	3919.27	4705.12	3919.27	4703.12	3886.83
Forest land	24,887.55	25,176.17	23,723.93	23,985.54	23,189.20	24,867.71
Grassland	4198.31	3909.57	4486.15	5037.97	5037.97	4181.84
Water	311.17	311.17	373.4	373.4	373.4	350.50
Residential land	136.49	136.49	153.27	136.49	143.27	155.24
Industrial and mining transportation land	28.56	28.56	39.36	28.56	34.27	39.09

## Data Availability

The data are available in a publicly accessible repository that does not issue DOIs. The land use data of Hechi City used in this study were obtained from the 30×30 m raster data of the Resource and Environment Science and Data Center of the Chinese Academy of Sciences (http://www.resdc.cn/, accessed on 27 May 2022).
